# A Visit to the Massachusetts State Sanatorium at Rutland

**Published:** 1907-09

**Authors:** Henry R. Slack

**Affiliations:** President Georgia Pasteur Institute, Atlanta, Superintendent LaGrange Sanatorium, LaGrange, Ga.


### A VISIT TO THE MASSACHUSETTS STATE SANATORIUM AT RUTLAND.*

By Henry R. Slack, Ph. M. D., President Georgia Pasteur Institute, Atlanta, Superintendent LaGrange Sanaforium, LaGrange, Ga.

Last summer while a student at Harvard Medical School, I rereceived an invitation from Dr. Cabot to visit the Massachusetts State Sanatorium for incipient tuberculosis at Rutland. It is needless to say that the invitation was gladly accepted, especially as I had a previous one from Dr. Edward O. Otis of Boston, one of the two examining physicians, and from Dr. Walter J. Marcley who had been superintendent since the establishment of this, the oldest of state institutions for the care of incipient tuberculosis.

About twenty of the class in physical diagnosis met Drs. Otis and Cabot at North Station and after a ride of nearly two hours through the roughest part of Massachusetts, we reached Rutland, which is very near the center of the state and the highest inhabited point.

At the station we were met by a number of conveyances that carried us over a pretty hilly country direct to the Sanatorium. There we were given a hearty welcome by the superintendent, who after registering each of us showed us all over the institution from the kitchen to the chapel.

It is constructed on the block plan and consists of sixteen buildings extending fan-like towards the south, thus securing the maximum of sunshine, and reducing the cold north winds to a minimum.

The buildings have a large number of windows in them, that are kept open night and day, winter and summer. The only time they are closed is between 6 and 7 A. M., when the steam heat is turned on, the rooms made comfortable, and the patients required to take cold baths ,either sponge or shower, and dress for the day. At first the patients seriously object to this, but they soon learn that instead

*Read before the Georgia Anti-Tuberculosis League Atlanta, Augnst 3d, 1907.

of it giving them more cold, it renders them less susceptible to cold, and they cheerfully acquiesce.

The first thing that impresses one on entering is the cleanliness and thorough system, then the air of hopefulness that pervades every ward. The patients were all "Spinners in the sun." After going through about half the wards we were invited to dinner which we were assured was a sample of their regular noon meal. The following was the menu: Lamb chops, creamed potatoes, boiled rice, stewed tomatoes, boiled corn, bread, milk, butter, ice cream, ftuit and coffee. Every article was well cooked and nicely served. With such diet as that, combined with sweet milk and raw eggs between meals, there is little surprise that 782 of the patients gained on an average 13 1-2 lbs. while only 57 lost weight. The wonder is that they have been able to keep the cost of living down to $9.38 per week, each.

After lunch we were taken to the amusement hall where we examined over 200 cases of incipient tuberculosis. Some of these cases were more advanced than I expected to find in such an institution and I was told that the confidence of the physicians in managing the disease had increased in the last few years so, that they now admitted more advanced cases than at first.

The report of the visiting physicians for 1906 shows 74.4 per cent. of apparent cures in incipient cases. All showed improvement except about 8.8 per cent.

This is a sanatorium in the true sense of the word, i. e., a place where people go to recover by learning the rules of health, hygiene, and sanitation. They are taught that consumption is not an inherited disease, but is usually contracted from "germs," contained in the sputum of consumptives. It is, therefore, very necessary to destroy the sputum, burning it up and not expectorating it on the floor, street, or elsewhere to dry and fly around in the dust to infect others. That incipient tuberculosis is a curable disease if they will follow the advice of a good physician and take plenty of nourishing food, fresh meat, raw eggs, sweet milk, butter, oatmeal, rice, and other vegestables. They must also breathe fresh air deeply through their nostrils night and day, summer and winter, spend their days in the sunshine or open air and sleep with their widows up winter as well as summer. They must avoid all fatiguing exercise; that daily baths in a warm room are healthful, live a regular life, keep the bowels open and get plenty of sleep. Keep clean company and a clear conscience. Avoid all alcoholic drinks, quack medicines, or "Consumption cures."

It is very interesting to note how earnestly the patients enter into the work of getting well, and how the older patients watch and teach the newer ones the rules and see that they obey them.

While this institution is supported by the state every patient is required to pay $4.00 per week toward his support. The managment assured me that this added very much to the success of the treatment and to the interest the patients took in carrying out instructions.

I think if Georgia succeeds in establishing such an institution, it will be well for the superintendent to spend some time at Rutland studying their methods.

It may be of interest since the passage of the Hardman-Covington-Neil prohibition bill to know that this is practically a temperance institution. To quote from the last report of the visiting physicians: "Contrary to old customs, the use of alcohol has been abolished, except in rare instances. We are sure that alcohol is one of the most common causes of tuberculosis and believe that the disease can be better treated without it. Even the old time honored cod liver oil has fallen into disrepute with us, being given in only about one case out of an hundred. Good common food well cooked answers the purpose much better; and while be believe in generous supplies of nourishing food we do not believe in the indiscriminate and unlimited stuffing which is advocated by some, and from which we think there is now a perceptible reaction, as its disadvantages become more manifest."

For some years I have been following this line of treatment and the success that has crowned my efforts has been very gratifying. I usually have a frank talk with the patient and tell them they have tuberculosis, but that in its incipiency it is curable. Caution them about the danger of infecting others by being careless about their sputum, etc. Unless they realize this, they will not carry out instructions and I observe, the chances for recovery are much better in those who are bold enough to "Face the lion in his den," than in those who say, "Doctor, if my lungs are affected I don't want to know it. I have just a heavy cold and want something for my cough." To these death comes "in consumption's ghastly form," and to check his stealthy insiduous advance we are here to plead for a state institution to teach our citizens how to protect themselves against this "Captain of the Hosts of Death."

